# Pathway to care and time to treatment among patients attending psychiatry service at Dilla, Ethiopia, a cross-sectional study

**DOI:** 10.1371/journal.pmen.0000298

**Published:** 2025-09-10

**Authors:** Misrak Negash Shonor, Rediet Dereje, Chalachew Kassaw, Solomon Moges Demeke, Biazin Yenealem, Yohanes Sime, Tadese Teferi

**Affiliations:** 1 Department of Psychiatry, College of Medicine and Health Science, Dilla University, Dilla, Ethiopia; 2 Department of Psychiatry, College of Medicine and Health Science, Woldia University, Woldia, Ethiopia; PLOS: Public Library of Science, UNITED KINGDOM OF GREAT BRITAIN AND NORTHERN IRELAND

## Abstract

Mental health disorders are a significant global health concern. Timely access to psychiatric care is crucial for positive treatment outcomes. However, the path to care followed can vary greatly. Understanding these pathways is essential for identifying potential delays in accessing treatment. Therefore, this study aimed to assess the pathway to care and the time to treatment among patients attending psychiatry services. The cross-sectional study was conducted from July to October 2023. A systematic random sampling technique was employed to recruit a sample of 424 participants. Data were collected using questionnaires. The Epi-collect smartphone application was used to collect data and then data was exported and analyzed using SPSS version 26. Bivariate and multivariate logistic regression analyses were conducted to identify factors associated with paths to psychiatric care. Statistical significance was set at p < 0.05. The magnitude of direct pathway to care, and delayed treatment was 5.9% (95% CI: 3.9-8.6%) and 51.7% (95% CI: 46.8-56.5%) respectively. Several factors were associated with the direct pathway to care. Younger adults (AOR: 2.80, 95% CI: 1.384, 7.276), males (AOR: 3.0, 95% CI: 2.048, 6.037), and those with schizophrenia (AOR: 2.4, 95%CI: 1.6, 4.8) were more likely to use direct pathway to psychiatry care. In contrast, Poor social support, low mental health literacy, lack of awareness about treatment availability and greater distance to a health facility were associated with a decreased likelihood of taking direct pathway to psychiatric care. This study found limited use of direct pathways to psychiatric care. Poor social support, low mental health literacy, lack of awareness about treatment availability and greater distance to a health facility were associated with a decreased likelihood of taking a direct pathway to psychiatric care.

## Background

Mental health disorders are a significant global public health concern, affecting millions of individuals worldwide. These disorders are characterized by alterations in thinking, mood, and behavior, leading to distress and impaired functioning in daily life [1]. The World Health Organization (WHO) estimates that one in four people globally will experience a mental health condition, and in Africa, over 80% of the population seek healthcare from traditional healers, with 40–60% specifically for mental health problems [2,3].

People with different mental health conditions went through varied paths to access mental healthcare. The events, processes, and intervals preceding medical treatment are collectively known as pathways to care [4]. These pathways typically originate within a specific social context, triggered by culturally influenced help-seeking interactions between individuals experiencing distress and their significant others [5]. Psychiatric care pathways can be either direct or indirect. Direct path involves receiving treatment directly from psychiatrists or mental health professionals without consulting alternative sources. Conversely, in-direct path entails seeking help from psychiatrists or mental health professionals after exploring alternative options.

Studies conducted in high-income countries indicates that a significant proportion of individuals with mental disorders directly access mental health services from psychiatrists or mental health professionals in clinics or hospitals [6–8]. For instance, 33.8% of study participants reported direct access to mental health care in study conducted in Italy [9], 49, 6% of participants were went directly to the psychiatric emergency department for help without contacting any other care providers in Lisbon’s study [6], In contrast, African studies revealed that over half of participants initially turn to traditional and religious healers for care [10–13]. For instance, in Nigeria, one study reported that 39.9% of participants first contacted traditional or religious healers when they became mentally ill [14], while another found that 48.1% of the pooled proportion of participants initially sought help from informal providers for mental disorders [15]. Furthermore, a study conducted in Ghana revealed that 48% of patients initially contacted non-psychiatric treatment centers (faith-based, traditional healers, and general medical practitioners) as their first point of contact for mental disorders [16]. Similarly, a study from Ethiopia showed that 71.4% of patients with mental illness first sought help from religious healers [17] and in the northern part of Ethiopia, among people with depressive symptoms, 14.3% sought treatment from healthcare settings, 15.5% from non-healthcare settings, and 19.6% from any source ( [18].In low-income countries, individuals with mental disorders often experience prolonged periods of untreated illness [17,19]. Several factors contribute to this delay, including the prioritization of traditional and faith healers as primary sources of help [20], inadequate mental health infrastructure [21], limited awareness of available mental health services [22], and the perception of mental illness as insignificant [23]. Delayed access to psychiatric care can exacerbate distress and disability, and hinder early detection, identification, and intervention of mental disorders [24,25].

Multiple studies have explored factors significantly associated with pathways to psychiatric care. A study conducted in Portugal, showed that direct pathways were significantly linked to male gender, involuntary admission, referral by a family member, lower household size, and fewer prior contacts with mental health services. In contrast, an study from Italy demonstrated a significantly lower self-referral rate to psychiatric care among individuals with schizophrenia (40.9%) compared to those with affective (73.57%), neurotic (87.85%), or eating disorders (81.25%) [9]. A study from England, showed that younger age and suicidal ideation were significantly associated with shorter direct pathways to psychiatric care. Conversely, older age, marital status, somatic symptoms, and anxiety and depression diagnoses were linked to longer pathways [26]. These studies collectively suggest that demographic and clinical characteristics can influence decisions regarding direct or indirect pathways to psychiatric care.

Studies conducted in low- and middle-income countries (LMICs) showed that several factors influenced the preference of individual choices of care. A study conducted in Nigeria, found that higher education predicted preference for the biomedical model. In contrast, low education was associated with traditional and spiritual pathways. In terms of religion, Protestants preferred the spiritual path more than Catholics [21]. Another qualitative study conducted in the Delta region of Nigeria found that the reason for choosing indirect pathways was influenced by religious beliefs about treatment (such illness can be cured only by the power of God), traditional beliefs about the causality of mental illness (handwork of witches, spiritual attacks such as `black magic’ and `evil spirits’), lack of knowledge about availability of mental health service, and stigma and discrimination [22].

We found few studies conducted on pathways to psychiatric care in Ethiopia [27]. Those studies were conducted in different settings than the one we conducted, i.e., they were conducted at Butajira [28], Jimma [29], and Addis Ababa [27]. Most of the studies were conducted ten years ago, when psychiatric care was not widely available, and the numbers of mental health professionals were scarce. Therefore, the purpose of this study was to determine the pathway to psychiatric care and its associated factors among people attending psychiatric service at Dilla University General Hospital.

## Methods

### Study design and setting

The cross-sectional study was conducted between July 2023 and October 2023 at Dilla University General Hospital. The hospital provides medical services to ~5 million people in the southern parts of Oromia, SNNPR, and Somalia and is situated 365 km south of the capital city, Addis Ababa. The psychiatry service was started in 1978 G.C., and ~400 patients attend mental health services monthly, according to the hospital’s HMIS report of mental health services.

### Population

This study considered a source population encompassing all patients receiving ongoing psychiatry care at Dilla University General Hospital. This included both inpatients (admitted to the hospital) and outpatients (receiving follow-up care). The study population consisted of all patients on follow-up and in-patient units during the data collection period. Furthermore, to ensure accurate diagnoses, only patients with confirmed Mental Disorders based on the DSM-5 classification system were included. Confirmation was made by a senior mental health professional with a Master of Science degree in Psychiatry working at Dilla University General Hospital.

### Eligibility criteria

The study participants whose age is greater than or equal to 18 years old, with a confirmed diagnosis of at least one mental disorder according to DSM-5, receiving treatment at the Mental Health Service of Dilla University General Hospital and willingness to provide informed consent were included in the current study. Participants with impairment that could limit their understanding or ability to consent were not included. Similarly, those experiencing an acute mental health crisis or active psychosis were excluded, as this could affect the accuracy of their responses.

### Sample size determination

The sample size was calculated using a single population proportion formula, assuming a 95% confidence level. As no prior study had been conducted in the study area, a conservative estimate of the population proportion, p = 0.5, was used. With a desired margin of error, d = 0.05, the required sample size was calculated as follows:


$$n\;=\;(z\alpha /2{)}^{2}\;*\;p\;*\;(1-p)\;/\;d^{2}\;=\;384\mathrm{\backslash ]}$$

For possible non-response during the survey, a 10% non-response rate was added, resulting in a final sample size of 424 participants.

### Sampling technique and procedure

A systematic random sampling technique was employed to select participants. The total population was estimated to be 1200 psychiatric patients based on an average monthly visit rate of 400 to Dilla University General Hospital over three months. To ensure a representative sample, an interval of 3 was determined by dividing the total population by the desired sample size of 424. A random number between 1 and 3 was then drawn to randomly select the starting point, and every third patient thereafter was included in the sample.

### Study variable

The dependent variable was the pathway to psychiatric care, and the independent variables included various sociodemographic factors such as age, sex, marital status, religion, educational status, economic status, place of residence, and distance to a healthy facility. Clinical and psychosocial factors such as social support, perceived stigma, awareness about the availability of psychiatric services, perceived severity of illness, and diagnosis of illness based on DSM-5 were also considered independent variables.

### Data collection tool and procedures

**Path to psychiatric care**: to understand participants’ care journeys before reaching a mental health professional, this study employed a semi-structured interviewer-administered questionnaire. This questionnaire was informed by the concept of a ‘pathway encounter form.’ While there isn’t a universally used WHO form specifically for this purpose, we referenced the WHO’s collaborative ‘pathway study’ encounter form (1987) as a guiding framework [30]. The questionnaire gathered systematic data on the sources of care participants utilized across various healthcare providers, both formal and informal, in different countries. This encounter form was used in several studies [17,31].

**A direct pathway to psychiatric care**: involves an individual with a suspected mental health disorder initiating contact with a qualified mental health professional, such as a psychiatrist, psychologist, or psychiatric nurse practitioner, for assessment and treatment. This can involve scheduling an appointment at a private practice, clinic, or hospital, or visiting an emergency room in a mental health crisis for evaluation and potential referral.

**An indirect pathway to psychiatric care**: involves seeking initial assistance from individuals other than qualified mental health professionals, such as traditional healers, religious leaders, or primary care physicians, who may then refer the individual to mental health specialists. Additionally, individuals can access mental health hotlines or online screening tools for resources and support without initially contacting a mental health professional directly.

For this study, we categorized the path to psychiatric care as follows: **Direct pathway:** Individuals who directly sought help from a mental health professional after experiencing a mental health issue [32]. **Indirect pathway:** Individuals who initially sought help from other sources, such as religious leaders, general practitioners, or traditional healers, before reaching a mental health professional. Studies in Ethiopia have employed a similar assessment approach.

**The Oslo Social Support Scale (OSSS-3)** was employed to measure social support [33]. The OSSS-3 assigns a total score ranging from 3 to 14. Scores of 3–8 indicated poor social support, scores of 9–11 indicated moderate support, and scores of 12–14 indicated strong social support. Prior studies has demonstrated lower end acceptable internal consistency for the OSSS-3 (α = 0.640). Notably, this tool has also been successfully utilized in previous studies conducted within Ethiopian settings [34–36].

**Time to treatment**: was considered delayed if the reported duration of untreated illness exceeded the median total duration reported in the sample [17,37].

**Awareness about the availability of mental health service:** To assess awareness of psychiatric treatment availability, a single item with a “yes” or “no” option was used. Similar assessments have been used in Ethiopian studies..

**Mental health literacy**: The 36-item Mental Health Literacy Tool (MHLT-36) is a valuable tool for researchers seeking to assess individuals’ understanding of mental health. This standardized tool delves into a person’s overall knowledge, attitudes, and beliefs about mental illness [38]. The MHLT-36 can be used to evaluate different aspects of mental health literacy. For instance, it includes items that test the ability to recognize symptoms of common mental disorders like depression, anxiety, and schizophrenia. It also assesses basic knowledge about causes, treatments, and prognosis of mental illness. While not directly measuring help-seeking behavior, the MHLT-36 can indirectly gauge attitudes toward seeking professional help for mental health concerns. Scoring is straightforward, utilizing a four-point Likert scale where higher scores reflect greater mental health literacy. A key strength of MHLT-36 is its established reliability and validity across diverse populations [39–41]. Additionally, it offers a comprehensive assessment of various mental health literacy aspects within a single, convenient tool. Certain studies in Ethiopia have employed a similar assessment approach [42–45].

**Perceived Stigma Scale (PSS-4)**. Was assessed by the Perceived Stigma Scale (PSS-4) [46]. This is a tool used to measure an individual’s perception of negative attitudes and beliefs towards people with mental illness. The PSS-4 consists of four core items that participants rate on a Likert scale, indicating their level of agreement. Examples of statements include whether people with mental illness are dangerous or unable to hold jobs. Some statements are reverse-scored, meaning strongly disagreeing translates to a higher score. After reverse scoring these items, a total score is calculated by summing the scores across all four statements. Higher total scores on the PSS-4 reflect a stronger perception of the stigma associated with mental illness. The PSS-4 has been established as a reliable and valid tool in various research settings. Some studies in Ethiopia have employed a similar assessment approach.

### Data quality control

The questionnaire was initially prepared in English and then translated into Amharic and Gedeuffa, the local language of the Gedeo Zone. To guarantee consistency and clarity, the translated versions were back-translated into English by two experts. Pre-testing was conducted on 5% of the sample size at Hawassa Comprehensive Specialized Hospital. The feedback obtained from this pre-test was used to develop the final version of the questionnaire. Data collectors and supervisors received training from the principal investigator on the questionnaire itself, data collection methods, quality control measures, and ethical considerations. The questionnaire’s reliability and participant understanding were assessed. During data collection, site supervisors provided oversight. Once the data collection process was complete, the completed questionnaires were checked for completeness and consistency.

### Data processing and analysis

The collected data was collected by using the Epi-Collect Smartphone application. It was then exported to SPSS version 26 for analysis. Crude and adjusted odds ratios were used to measure the association between independent variables and pathways to psychiatric care. Chi-square tests were employed for categorical independent variables to assess assumptions. Results were presented using frequency tables and charts. The normality of the data was checked. Descriptive statistics were presented using means and standard deviations for normally distributed numerical data. Both multivariable and bivariable logistic regression models were used to assess the association of independent variables with the dependent variable. Variables with a p-value of ≤0.025 in the bivariable logistic regression analysis were selected for inclusion in the multivariable logistic regression analysis. Variables with a p-value of <0.05 in the multivariable logistic regression analysis were declared as statistically significant in the pathway to psychiatric care.

### Ethical statement

Ethical approval was obtained from the Institutional Review Board (IRB) of Dilla University College of Medicine and Health Science before conducting the study. Written consent was obtained from each study participant before the start of the data collection. Personal identification was kept confidential throughout the study. Participants were also assured that they could withdraw from the study at any point if they wished.

## Result

### Sociodemographic characteristics of study participants

A total of 424 participants with the diagnosis of different mental disorders were included in the study, resulting in a response rate of 100%. The majority of participants were in the age range between 31 and 40 years, comprising (234; 30.4%) of the total sample. More than half were female (221; 52.1%), protestant in religion (266; 62.7%), completed high school (228; 53.8%). The monthly income of most of the respondents was in the range of 1001–3000Birr (225; 88.6%), and the majority working as daily laborers (93; 21.9%) (Table 1).

**Table 1:** Sociodemographic characteristics of study participants at Dilla University General Hospital, Gedeo Zone, South Ethiopia Region, 2023.

Variable	Category	Frequency	Percent
Age(in year)	18-30	79	9.3
	31-40	234	30.4
	41-50	85	15.9
	>=51	26	5.8
Sex	Male	203	47.9
	Female	221	52.1
Religion	Orthodox	100	23.6
	Muslim	53	12.5
	Protestant	266	62.7
	Other	5	1.2
Educational Status	Unable to read and write	18	4.2
	Elementary school	155	36.6
	High school	228	53.8
	Degree and above	23	5.4
Occupational status	Jobless	34	8.0
	Daily laborer	93	21.9
	Farmer	85	20.0
	Private business	62	14.6
	Student	28	6.6
	Housewife	86	20.3
	Civil servant	36	8.5
Monthly income	0-1000birr	170	40.1
	1001-3000birr	225	88.6
	>3001birr	29	6.8

### Distribution of path to mental healthcare among study participants

In terms of first contact with psychiatric care, the majority (208, 49.1%) of participants pursued treatment based on recommendations from family members. Among those studied, 48 (11.3%) came with a referral letter, while 118 (27.8%) had a previous history of utilizing mental health services. Among the study participants, 188(44.3%) were diagnosed with epilepsy, followed by 141(33.3%) who were diagnosed with schizophrenia. Of the study participants, 25(5.9%) reached out to psychiatric services, whereas the majority, 191(45%), sought help from religious leaders, and 153(36.1%) looked for help from traditional healers (Table 2).

**Table 2:** Distribution of path to mental healthcare among patients attending mental health services at Dilla University General Hospital, Gedeo Zone, South Ethiopia Region, 2023.

Variables	Category	Frequency	Percent
Who recommended a healthcare provider	Patient himself	44	10.4
	Former patient	81	19.1
	Family	208	49.1
	*Others	91	21.5
Does the patient have a referral letter	Yes	48	11.3
	No	376	88.7
Had the patient received mental health services in the past	Yes	118	27.8
	No	306	72.2
Diagnosis based on DSM-5	Other psychotic disorder	43	10.1
	Schizophrenia	141	33.3
	Major depressive disorder	23	5.4
	Bipolar Disorder	19	4.5
	Epilepsy	188	44.3
	Anxiety	10	2.4
Where did you receive care	Traditional healer	153	36.1
	Psychiatric service	25	5.9
	Religious leader	191	45.0
	General practitioner	55	13.0

*Others include health professionals, work mate and neighbors.

### Mental healthcare seeking and the perceived reasons behind these behaviors

This study assessed how participants sought mental healthcare and the perceived reasons behind these behaviors. Of the study participants, 208(49.1%) sought help through a recommendation from a family member or relative, followed by those 86(20.3) who looked for recommendations from former patients who had received treatment. Out of the participants, the majority, 188(44.3%), mentioned worsening of illness as the main reason for seeking help, while functional impairment was the second reason mentioned. The majority of participants, 167(39.4%), cited a lack of knowledge about where to find help as a reason for not seeking help, while a close second were those 158 (37.3%) who mentioned financial difficulty. Furthermore, of those studied, 149(35.1%) mentioned the evil eye as the perceived cause of mental illness, followed by 142(33.5%) participants who mentioned spiritual possession as the perceived cause of mental illness. Finally, among the study participants, 191 (45%) initially sought care from a religious leader, while 153 (36.1%) turned to a traditional healer as their first point of contact (Table 3).

**Table 3:** Distribution of contacts of care among patients attending mental health services at Dilla University General Hospital, Gedeo Zone, South Ethiopia Region, 2023.

Variables	Category	Frequency	Percent
Who recommended that you seek care?	Neighbor	48	11.3
	Family/relative	208	49.1
	Friends	46	10.8
	Patient himself	26	6.1
	Former patient	86	20.3
	Health professional	10	2.4
What was the main problem	Suicidal behavior	63	14.9
	Aggressive behavior	25	5.9
	Functional impairment	148	34.9
	Worsening illness	188	44.3
Reasons for not seeking care sooner	Financial difficulties	158	37.3
	Didn’t know where to seek help	167	39.4
	Lack of mental health service	47	11.1
	Distance	52	12.3
Perceived causes of mental illnesses	Evil eye	149	35.1
	Sinful act	77	18.2
	Stress	5	1.2
	Spiritual possession	142	33.5
	Family history	20	4.7
	I don’t know	31	7.3
Where did you receive care	Traditional healer	153	36.1
	Psychiatric service	25	5.9
	Religious leader	191	45.0
	General practitioner	55	13.0

### Mental illness perception of study participants

This study assessed participants’ perceptions of mental health. Of the study participants, 279 (65.8%) stated that there is no cure for mental illness, followed by 130 (30.7%) who were unsure about the curability of mental illness. Among the study participants, 179(42.2%) referred to church prayer or exorcism as a treatment for mental illness, while the second mostly mentioned being holy water service. Of those studied, 176(41.5) claimed that people who use drugs become mentally ill, while the second mostly cited being people with crises. Furthermore, the majority of study participants, 270(63.6) were perceived mental illness as highly severe. Finally, out of participants, 185(43.6%) perceived mental illness as highly shameful (Table 4).

**Table 4:** Mental illness perception of study participants at Dilla University General Hospital, Gedeo Zone, South Ethiopia Region, 2023.

Variables	Category	Frequency	Percent
Are mental illnesses curable?	Yes	15	3.5
	I am not sure	130	30.7
	No	279	65.8
Which treatment can be used to treat mental illness?	Mental health professional	30	7.1
	Church for prayer/Exorcise	179	42.2
	Holy water	84	19.8
	Traditional healer/herbalist/	63	14.9
	Traditional healer/witchcraft	68	16.0
Which kinds of people are affected by mental illnesses?	People with crisis	103	24.3
	Angry and stressed	67	15.8
	People who use drugs	176	41.5
	Those who think a lot	58	13.7
	Others	20	4.7
Perceived severity of mental illnesses	Less severe	78	18.4
	Severe	62	14.7
	Highly severe	270	63.6
	Very highly severe	14	3.3
Perception of mental illness	Very highly shameful	92	21.7
	Highly shameful	185	43.6
	Shameful	128	30.2
	Not as such shameful	14	3.3
	Not at all shameful	5	1.2

### Paths to psychiatric care taken by study participants at Dilla University General hospital

**Fig 1:**
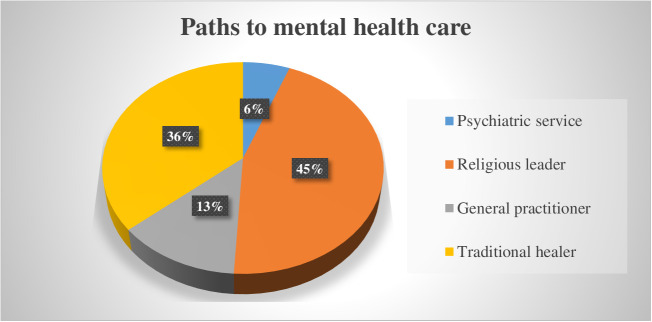
Showing paths to psychiatric care taken by study participants at Dilla University General Hospital, Gedeo Zone, South Ethiopia Region, 2023.

Study participants mentioned different paths they went through as their first point of contact in their looking for help. The majority of participants, 45.0%, reported a religious leader as their first point of contact for help, followed by those (36.1%) who went to traditional healers. Out of the study participants, 5.9% of participants sought help from a psychiatric service. These findings suggest that traditional healers and religious leaders may be a more common first point of contact for mental health care than formal mental health services. (Fig 1).

### The magnitude of a direct path to psychiatric care and delayed treatment

**Fig 2:**
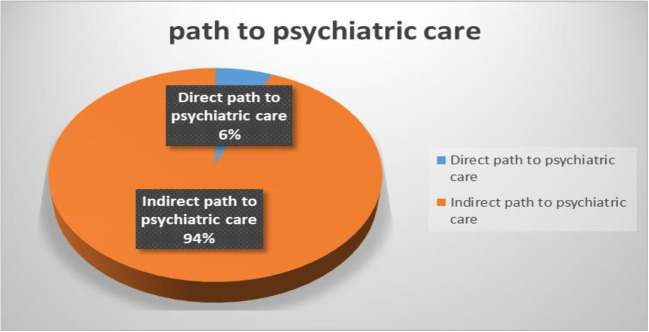
Showing the magnitude of paths to psychiatric care among study participants at Dilla University General Hospital, Gedeo Zone, South Ethiopia Region, Ethiopia, 2023.

**Fig 3:**
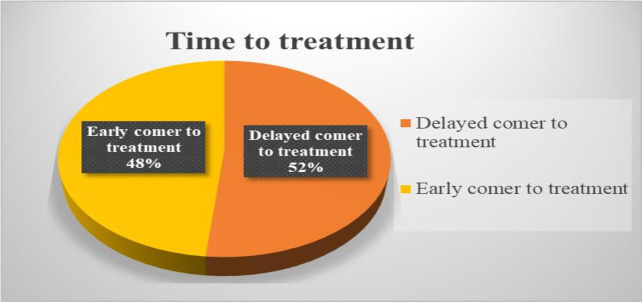
Showing the magnitude of Time to treatment among study participants at Dilla University General Hospital, Gedeo Zone, South Ethiopia Region, Ethiopia, 2023.

This study assessed the magnitude of a direct path to psychiatric care and associated delays in seeking psychiatric service for mental health concerns. The magnitude of the direct pathway to care, and delayed treatment was 5.9% (95% CI: 3.9-8.6%) and 51.7% (95% CI: 46.8-56.5%) respectively (Fig 2 and Fig 3)

### Factors associated with paths to psychiatric care

In the current study several factors were associated with a direct path to psychiatric care, including younger age, male gender, having a diagnosis of schizophrenia or bipolar disorder, and educational status. Study participants whose age ranged from 18 to 30 years old were more likely to directly seek out psychiatric care when compared to older individuals (AOR: 2.80, 95% CI: 1.384, 7.276, p-value = 0.01). Participants with male gender were three times more likely to take a direct path than females (AOR: 3.0, 95% CI: 2.048, 6.037). Those participants with the diagnosis of schizophrenia, bipolar disorder, and those perceiving mental illness as highly or very highly severe were more likely to take a direct path (AOR: 2.4, 2.3, 2.52, and 4.00 respectively, all with p-values < 0.01). Those study participants unable to read and write were 80% less likely to take a direct path to psychiatric care compared to participants with a degree (AOR: 0.20, 95% CI: 0.089, 0.796). Similarly, social support, mental health literacy, and income were associated with a direct path to psychiatric care. Participants with poor social support, low mental health literacy, or a monthly income below 1000 ETB were less likely to take a direct path to psychiatric care when compared to their counterpart. Compared to their counterparts, the chance of directly seeking psychiatric care decreased by 97% for those with poor social support (AOR: 0.03, 95% CI: 0.012, 0.109), 86% for those with low mental health literacy (AOR: 0.14, 95% CI: 0.075, 0.386), and 90% for those with lower income (AOR: 0.10, 95% CI: 0.065, 0.280). Furthermore, those aware of treatment availability were more likely to take a direct path (AOR: 1.4, 95% CI: 1.133-2.282) compared to their counterparts. Study participants with low perceived stigma were more likely to take a direct path compared to those with high perceived stigma (AOR: 2.0, 95% CI: 1.602-3.205). Finally, participants living closer to a health facility (<5km) were more likely to take a direct path compared to those dwelling in more than 5km from a health facility (AOR: 2.10, 95% CI: 1.684-3.328) (Table 5).

**Table 5:** Description of Bivariable and multivariable binary logistic regression analysis showing an association between pathway to psychiatric care and associated factors among study participants at Dilla University, Gedeo zone, South Ethiopia Region.

Variables	Category	Path to psychiatric care		COR(95% CI)	AOR(95% CI)	P-Value
		Direct	Indirect			
Age(in year)	18-30	66	13	3.17(1.180, 8.530)	2.80(1.384, 7.276)*	0.01
	31-40	4	230	0.01(0.003, 0.039)	0.01(0.004, 0.031)*	0
	41-50	5	80	0.04(0.012, 0.130)	0.02(0.014, 0.107)*	0
	>=51	16	10	1		
Sex	Male	39	164	3.5(1.846, 6.696)	3.0(2.048, 6.037)*	0.000
	Female	14	207	1	1	
Educational Status	Unable to read and write	6	12	0.26(0.072, 0.981)	0.20(0.089, 0.796)*	0.02
	Elementary school	90	65	0.73(0.296, 1.845)	0.50(0.343, 1.592)	0.25
	High school	104	124	0.44(0.182, 1.097)	0.37(0.211, 0.949)	0.03
	Degree and above	15	8	1	1	
Diagnosis based on DSM-5	Schizophrenia spectrum disorder	70	53	2.83(1.498, 5.382)	2.4(1.666, 4.856)*	0.000
	Major Depressive disorder	1	16	0.13(0.017, 1.085)	0.10(0.023, 0.776)	0.02
	Bipolar Disorder	14	9	2.79(1.049, 7.450)	2.3(1.228, 6.363)*	0.01
	Epilepsy	7	192	0.07(0.031, 0.197)	0.06(0.036, 0.170)	0
	Anxiety Disorder	20	4	1	1	
Social Support	Poor social support	12	278	0.035(0.010, 0.135)	0.03(0.012, 0.109)	0
	Intermediate Social support	15	108	0.11(0.031, 0.426)	0.10(0.036, 0.346)	0.000
	Strong social support	6	5	1	1	
Mental health literacy level	Low	5	200	0.17(0.065, 0.451)	0.14(0.075, 0.386)	0.000
	High	28	191	1	1	
Monthly Income	0-1000ETB	19	151	0.13(0.056, 0.322)	0.10(0.065, 0.280)	0.000
	1001-3000ETB	0	225	0.009(0.002, 0.046)	0.005(0.003, 0.036)	0.0
	>=3001ETB	14	15	1	1	
Perceived illness severity	Less severe	12	66	1	1	
	Severe	7	55	0.70(0.258, 1.9)	0.40(0.303, 1.618)	0.24
	Highly severe	107	163	3.61(1.863, 6.997)	2.52(2.072, 6.291)*	0.000
	Very highly severe	7	7	5.50(1.632, 18.534)	4.00(1.984, 15.246)*	0.00
Awareness about the availability of psychiatric treatment	Yes	90	150	1.6(1.060, 2.440)	1.4(1.133, 2.282)*	0.000
	No	50	134	1	1	
Perceived Stigma	High	50	134	1	1	
	Low	110	130	2.26(1.502, 3.425)	2(1.602, 3.205)	0.000
Distance from health facility	>=5km	54	130	1	1	
	<5km	119	121	2.37(1.578, 3.553)	2.10(1.684, 3.328)	0.000

Hosmer and Lemeshow test result was p-value = 0.65, * indicating factors with significant association.

## Discussion

This study assessed pathways to care and associated time to treatment among patients attending psychiatric services at Dilla University General Hospital. As per this study, the magnitude of the direct pathway to care and delayed treatment was 5.9% (95% CI: 3.9-8.6%) and 51.7% (95% CI: 46.8-56.5%), respectively. Several factors were associated with the direct path to care including, younger age, male gender, those with diagnoses of schizophrenia or bipolar disorder, perceived illness severity, educational status, social support, low mental health literacy, income status, lack of awareness about treatment availability, and distance to a health facility.

This study found that 5.9% of participants sought psychiatric services directly. This finding was comparable to the 9.2% rate reported in a study conducted in central India [47], suggesting that cultural or systemic factors common to both regions might be at play. However, the current study contradict the results of studies from Ghana and Ethiopia. A Ghanaian study showed that 52.3% of patients first sought care at psychiatric hospitals [16]. This study was conducted at Pantang Psychiatric Hospital in Accra, the capital city of Ghana. In one of Ethiopian study conducted at Ayder Comprehensive Specialized Hospital in Mekele city, the capital of Tigray Regional State, it was reported that 22.5% of study participants were used psychiatric service as first point of contact [17]. In another Ethiopian study conducted at Southwest Ethiopia, over a third of the patients (35.2%) came directly to psychiatric service [29]. Furthermore, a study conducted at Amanuel Mental Specialized Hospital in Addis Ababa, the capital city of Ethiopia, reported that 41% of participants were directly consulted a psychiatrist [27]. The differences could be due to study area and sample size. Studies conducted in capital cities tend to have larger proportions of patients using direct psychiatric services, which could be associated with greater accessibility to psychiatric services and higher literacy rates. Additionally, sample size could also play an important role, as the studies used different sample sizes ranging from 107 to 1044 [27,16].

The current study’s findings showed a gender difference in pathways to psychiatric care. Specifically, male participants were three times more likely to take a direct path to psychiatric care compared to females. This aligns with a study indicating that men might be less likely to report a broader range of symptoms, potentially leading to a situation where they appear to take a ‘direct path’ because they only seek help when their distress reaches a more severe level [48].

Furthermore, this study found that participants with severe mental disorders were more likely to take a direct path to care. This finding was consistent with a study that reported patients with Severe Mental Illness (SMI) often prefer immediate support from various mental health professionals in addition to, or even before, seeing a psychiatrist [49].

Moreover, the current study showed an association between mental health literacy and help-seeking behavior. Participants with lower literacy were 86% less likely to directly access psychiatric care. This finding aligns with a study where individuals with lower literacy were less likely to seek professional help for mental health concerns. To narrow this gap, interventions like educational programs or community outreach initiatives may empower individuals to recognize mental health concerns and navigate the path to appropriate care.

Additionally, this study found a significant association between social support and the path to psychiatric care. Participants with poor social support were 97% less likely to directly access help. This finding aligns with a study that reported fewer social supports were associated with a lower likelihood of utilizing mental health services after stressful events [50]. The likely reason for such an association is that patients with limited social support may feel isolated, lack the confidence to seek help, or simply not know where to begin.

The current study also found a positive correlation between awareness of mental health service availability and taking a direct path to care. This finding was consistent with a study suggesting that increased knowledge of mental health conditions and available treatment options can encourage help-seeking behavior [51]. Furthermore, this study found a positive association between low perceived stigma and directly seeking psychiatric services. Participants with lower stigma scores were more likely to take a direct path to care, aligning with a study that showed this correlation [52]. In addition, a study conducted in the northern part of Ethiopia showed that People with low levels of internalized stigma and positive attitudes towards mental illness were nearly threefold more likely to seek direct care from mental health professional [18]. Low stigma level acts as a facilitator because it reduces the fear of judgment and social isolation, major barriers to seeking help.

Finally, Current study showed an association between distance to mental health facilities and direct service utilization. Participants living closer to health facilities (within 5km) were more likely to take a direct path to care. This finding was supported by a similar finding from the study which reported increased use of psychiatric services when facilities are geographically accessible [53]. According to a systematic review, patients in low-income countries are often required to travel greater distances to reach healthcare facilities. This is primarily due to a scarcity of healthcare providers and difficult geographic conditions, which can exacerbate the challenges associated with accessing care [54]. Furthermore, a study conducted in Rwanda showed geographical inaccessibility as a significant obstacle to accessing mental health services [55]. These findings underscore the need for improved access to mental health care, especially in resource-constrained settings.

### Study limitation

This study’s findings may have limitations due to its design and recruitment methods. Selection bias is a concern as the study was conducted at a single General hospital. People who attend General hospitals may be more likely to be proactive about seeking help or have more severe mental illness compared to the general population with mental health concerns in Ethiopia, particularly those who wouldn’t travel long distances for care. Additionally, the study relied on self-reported data, which can be susceptible to recall bias and social desirability bias. Participants may have difficulty recalling details about their path to care or treatment initiation, or they may provide answers they believe are more favorable. Finally, the cross-sectional design allows investigators to identify associations between variables, but it cannot establish causation.

### Recommendation

Given the limited use of direct path to psychiatric services and associated delay in seeking mental healthcare we identified, this study suggests key recommendations to improve access and reduce treatment delays. First, targeted outreach and education programs focused on younger adults, males, and individuals with severe mental illness are crucial. These programs should prioritize mental health literacy, stigma reduction, and raising awareness of available treatment options. Second, initiatives that strengthen social support networks can significantly increase the likelihood of people directly seeking professional help. Third, addressing financial barriers, particularly for those with lower income through financial assistance programs, can significantly promote timely treatment initiation. Finally, both increasing the availability of mental health services in rural areas and actively combating stigma associated with mental illness is essential to encourage help-seeking behavior and direct access to psychiatric care.
